# Crystal structure of *Streptococcus pneumoniae* pneumolysin provides key insights into early steps of pore formation

**DOI:** 10.1038/srep14352

**Published:** 2015-09-25

**Authors:** Sara L. Lawrence, Susanne C. Feil, Craig J. Morton, Allison J. Farrand, Terrence D. Mulhern, Michael A. Gorman, Kristin R. Wade, Rodney K. Tweten, Michael W. Parker

**Affiliations:** 1 ACRF Rational Drug Discovery Centre, St. Vincent’s Institute of Medical Research, Fitzroy, Victoria 3065, Australia; 2 Department of Microbiology and Immunology, University of Oklahoma Health Sciences Center, Oklahoma City, Oklahoma 73104, USA; 3 Department of Biochemistry and Molecular Biology, Bio21 Molecular Science and Biotechnology Institute, The University of Melbourne, Parkville, Victoria 3010, Australia

## Abstract

Pore-forming proteins are weapons often used by bacterial pathogens to breach the membrane barrier of target cells. Despite their critical role in infection important structural aspects of the mechanism of how these proteins assemble into pores remain unknown. *Streptococcus pneumoniae* is the world’s leading cause of pneumonia, meningitis, bacteremia and otitis media. Pneumolysin (PLY) is a major virulence factor of *S. pneumoniae* and a target for both small molecule drug development and vaccines. PLY is a member of the cholesterol-dependent cytolysins (CDCs), a family of pore-forming toxins that form gigantic pores in cell membranes. Here we present the structure of PLY determined by X-ray crystallography and, in solution, by small-angle X-ray scattering. The crystal structure reveals PLY assembles as a linear oligomer that provides key structural insights into the poorly understood early monomer-monomer interactions of CDCs at the membrane surface.

*Streptococcus pneumoniae,* or pneumococcus, is the causative agent for a range of serious human diseases including pneumonia, bronchitis, bacterial meningitis, sepsis, otitis media and corneal ulcers^1^, some of which lead to morbidity and mortality around the world. Indeed, infection by *S. pneumoniae* accounts for a quarter of the deaths of young children in the developing world. The emergence of drug resistant pneumococci and the poor efficacy of pneumococcal polysaccharide vaccines have prompted the search for new vaccine and small molecule drug targets.

Pneumolysin (PLY), a major virulence factor of the bacterium^2^, is a pore-forming toxin belonging to the family of cholesterol-dependent cytolysins (CDCs). The toxin is an important candidate as a serotype-independent vaccine target against the bacterium. PLY is produced as a water-soluble monomer and recognises mammalian cells via its C-terminal domain (domain 4) before assembling into circular prepores of ~30–50 monomers on the surface of cholesterol-rich membranes^3^,^4^. The monomers in the prepore undergo critical structural changes, including the unfurling of two alpha helical bundles (α-HB1 and α-HB2) in domain 3 in each monomer that are then refolded into β-hairpins (transmembrane hairpins TMH1 and TMH2) for insertion into the membrane, which is facilitated by a structural collapse towards the membrane surface^5^,^6^. The resultant PLY β-barrel pore has a diameter of ~300 Å that causes lysis of the target cell^7^.

Domain 4 (D4) is the membrane-sensing domain of CDCs and, for many CDCs, membrane-bound cholesterol appears to be the receptor^8^. Two residues at the tip of D4 (Thr and Leu) have been identified as a recognition site for cholesterol^9^. CDCs contain a highly conserved undecapeptide region (ECTGLAWEWWR), called the Trp-rich loop^10^ that is also located at the tip of D4 and forms an interaction site with membranes^11^. Tryptophan fluorescence studies suggest cholesterol binds in a 1:1 complex with PLY and the cholesterol-binding site is close to the Trp-rich loop^12^. This loop is also a key element in the allosteric pathway that couples membrane binding in domain 4 to the conformational changes that have to occur in domain 3 for the conversion of the prepore to pore^13^. More recently, a small group of CDCs exemplified by the atypical CDC intermedilysin (ILY) have been shown to use a GPI-anchored protein called CD59, an inhibitor of complement pore formation, as a receptor^14^. Intriguingly, the membrane attack complex proteins involved in complement pores (eg. C8, C9) adopt similar three-dimensional folds to CDCs, although they lack a structural equivalent of D4^15^,^16^. In ILY, the interaction with cholesterol is still required for membrane insertion of the prepore complex^8^,^17^. The binding site for CD59 was identified to be on one face of D4 by mutagenesis studies^18^ that were subsequently confirmed by the crystal structure of ILY complexed to CD59^19^. Although all CDCs must form a cholesterol-dependent interaction to function, recent work on PLY suggests that it can bind yet another type of receptor: certain glycans including Lewis histo-blood group antigens^20^. In earlier work D4 had been implicated in binding to mannose in a dose responsive fashion by pull-down assays^21^. In the 2014 study the putative binding site for sialyl Lewis X (sLeX), the glycan with highest affinity, was suggested to be around the Trp-rich loop based on *in silico* analysis and supported by mutagenesis studies^20^.

In this paper we present the crystal structure of PLY and show that it adopts a very similar conformation in solution by small-angle X-ray scattering. The crystal structure reveals that PLY can form linear oligomers and PLY oligomers can also be formed in solution by raising the temperature to physiological values. The PLY structure shows the formation of linear head-to-head oligomers that are consistent with previous experimental data showing that PLY and PFO can be trapped at an early stage of interaction at the membrane surface by preventing domain 3 structural changes. This trapping results in the assembly of weakly interacting monomers that form linear oligomers. Hence, for the first time the crystal structure of PLY reveals details of early monomer-monomer interactions in PLY that are likely relevant to all CDCs.

## Results

### Crystal structure of PLY

**Figure 1:**
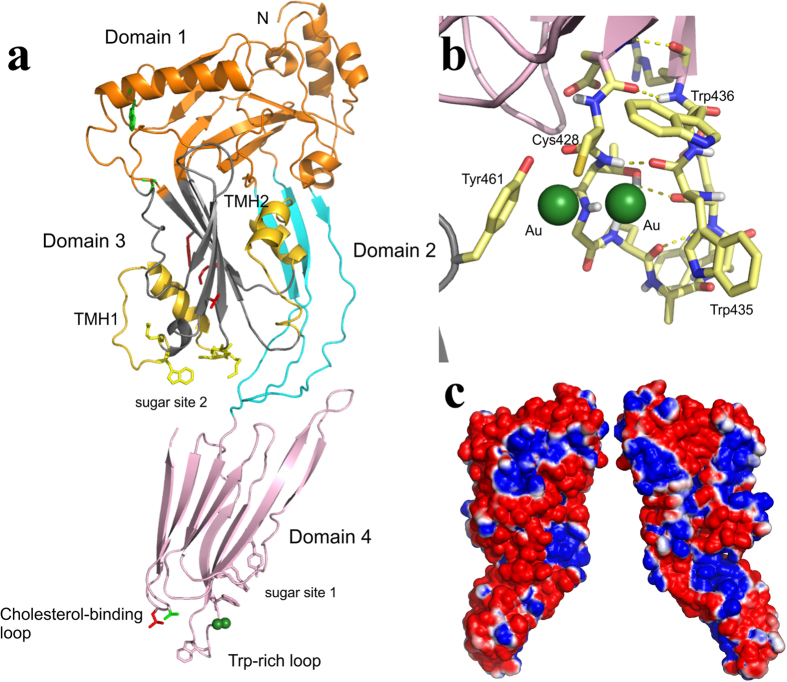
Crystal structure of the PLY monomer. (**a**) Ribbon representation of PLY. Domain 1 is coloured orange, domain 2 coloured cyan, domain 3 is coloured grey and domain 4 is coloured pink. The TMH1 and TMH2 regions are coloured yellow. The membrane-sensing loops of domain 4 (Trp-rich loop and cholesterol-binding loop) are indicated. The side-chains of residues involved in the putative sugar binding sites are highlighted with pink (Site 1) and yellow (Site 2) sticks. The residues corresponding to the electrostatic switch in PFO (Lys 152, Gly 166, Tyr 150, Val 287, Gly 305) are highlighted as red sticks in domain 3. The residues that have been shown to form linear oligomers when mutated in PFO (corresponding residue in PLY is W134) and PLY (Ala 146) are shown as green sticks in domain 1. (**b**) A zoomed in view of the gold binding sites (green spheres) in the Trp-rich loop. Tyr 461 is contributed from a symmetry related molecule. Also shown is the hydrogen bonding network (yellow dashed lines) in the Trp-rich loop region. (**c**) Electrostatic surface representation calculated using the program APBS^62^ in orthogonal views. Blue indicates electropositive potential (>1 kT) and red indicates electronegative potential (<−1 kT).

PLY adopts the canonical CDC fold^17^,^22^,^23^,^24^,^25^,^26^ with maximal dimensions of 123 Å by 20 Å by 49 Å. The molecule contains 11% helix and 32% beta-sheet. It is composed of four domains (Fig. 1a): domain 1 (D1; residues 1 to 21, 58 to 147, 198 to 243 and 319 to 342) consists of three α-helices and one β-sheet, domain 2 (D2; residues 22 to 57 and 343 to 359) contains one β-sheet, domain 3 (D3; residues 148 to 197 and 244 to 318) is composed of a 5-stranded anti-parallel β-sheet that is surrounded by the two α-helical bundles that become transmembrane hairpins TMH1 (residues 160 to 186) and TMH2 (residues 257 to 280), and domain 4 (D4; residues 360 to 470) is folded into a compact β-sandwich. Domain 2 is connected to domain 4 by a single glycine linker. Parts of domain 2 are highly mobile with ambiguous or weak electron density for some residues in these regions. Residues 268 to 280 on the α–HB2 (TMH2) loop in domain 3 and residues 306 to 312 in domain 3 also show relatively poor density. Two gold cyanide compounds, used to improve the quality of the crystals, are bound to Cys 428 (Fig. 1b). The location of the two gold compounds reflects alternative binding sites they can take up in the crystal. In one site the gold complex packs against Trp 435 and Trp 436 and the other packs against Tyr 461 from a symmetry related molecule. Despite these interactions the gold compound does not change the crystal packing as judged by comparison to an earlier lower resolution structure in which gold was not used.

### Comparison with other CDC structures

Available CDC crystal structures include PFO from *Clostridium perfringens*^22^,^27^, ILY from *Streptococcus intermedius*^17^, suilysin (SLY) from *Streptococcus suis*^24^, anthrolysin (ALO) from *Bacillus anthracis*^23^, streptolysin (SLO) from *Streptococcus pyogenes*^25^, and listerolysin (LLO) from *Listeria monocytogenes*^26^. The most significant difference between PLY and the other CDCs is that it adopts a linear shape down its long axis (Fig. 1a) whereas the other CDCs tend to be bent to various degrees due to different orientations of D4 with respect to the rest of the molecule. The closest homolog by sequence is SLY with a pairwise sequence identity of 52% (based on comparison of the mature forms for all CDCs where a sequence is available). Superposition of domains 1, 3 and 4 of the structure of SLY onto PLY gives root-mean-square (r.m.s.) deviations of the alpha carbon atoms of 1.5 Å, 1.5 Å and 1.1 Å, respectively. The low r.m.s values indicate that the 3D structures are generally very similar. A significant difference, not observed in any of the published CDC structures, is the high mobility of regions of two strands (residues 29 to 35, 46 to 50) in D2. Consequently, the hydrogen bond interactions that D2 forms with both D3 and D4 that are typically observed in SLY and other CDCs are absent in PLY.

The Trp-rich loop in PLY D4 (residues 427 to 437) adopts the same conformation as that previously seen in SLY and SLO, despite the binding of the gold complex to Cys 428. The only significant difference is that the gold binding has forced the side-chain of Trp 426 into solution. The hydrogen bonds between the residues in the Trp-rich loop are Glu 427 (O) to Arg 437 (N), Glu 427 (N) to Arg 437 (O), Thr 429 (OG1) to Glu 434 (O), Thr 429 (N) to Trp 435 (O) and Leu 431 (O) to Glu 434 (O) (Fig. 1b). There is one hydrogen bond to a symmetry related molecule [Cys 428 (O) to Tyr 461 (OH)]. Apart from Trp 463 in SLY and Trp 537 in SLO, which is a rotamer variation of Trp 435 of PLY, the hydrogen bonds within the Trp-rich loop are conserved in PLY, SLY and SLO. Additionally, PLY has a marked electronegative potential (Fig. 1c) in contrast to other CDCs but the reason for this is unclear.

We have recently shown in the archetypical CDC, perfringolysin O (PFO), that an intermolecular electrostatic switch controls the prepore-to-pore transition^28^. Monomers assembled into the prepore undergo a slight tilt, a process likely facilitated by an aromatic stacking interaction between Tyr 181 and Phe 318. This tilting positions Glu 183 (on the β1 strand) to form a salt bridge with Lys 336 (near β-strand 5) in the adjacent monomer of the prepore, an interaction that drives the coordinated disruption of the interface that domains 1 and 2 (D1,2) forms with one of the two α–HBs located in D3. Disruption of this interface (the D3-D1,2 interface) allows the unfurling of α–HB1, which then forms the membrane-spanning region TMH1. Mutation of either residue was shown to trap PFO in the prepore state, but the effect could be reversed by either weakening the domain interface by mutating Asn 197 or increasing the temperature. These residues are conserved in the majority of CDCs, but not in PLY (PLY contains a lysine at Glu 183, glycine residues at Lys 336 and Asn 197, and a valine at Phe 318), which suggested PLY coordinates the critical disruption of the D3-D1,2 interface by a different mechanism. Analysis of the PLY crystal structure provides insight in how its prepore-to-pore transition is facilitated: domain 2 is highly mobile and forms much fewer interactions with domain 3 compared to PFO, thus its interface is inherently less stable than the analogous structure in PFO. This is consistent with the observation that the thermal melting temperature (T_M_) of PLY, which is largely due to the disruption of the D3-D1,2 interface, is approximately 3 °C lower (46.2 °C, determined herein) than that previously found for PFO (49 °C)^28^.

### PLY forms an extended linear oligomer in the crystal

**Figure 2:**
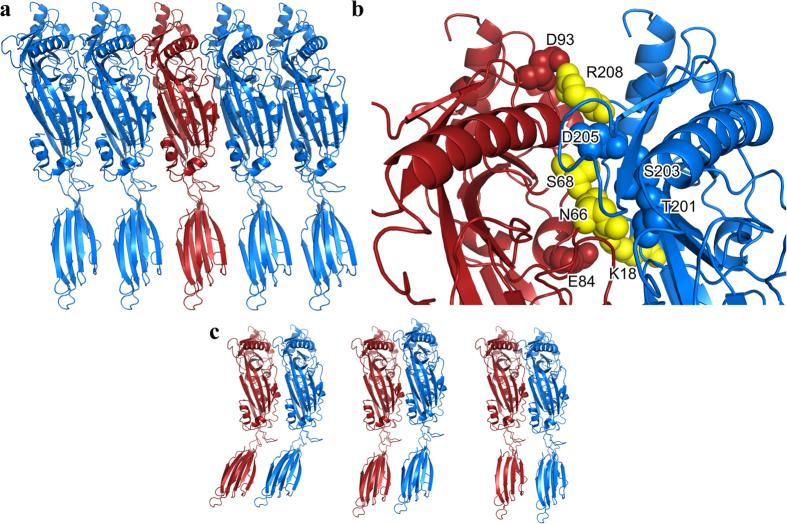
Oligomers in PLY crystals. (**a**) Crystal packing of PLY monomers. The monomers are arranged in a linear array. (**b**) Zoomed in view of the monomer-monomer interface in the PLY oligomer shown in panel (**a**) with residues that were mutated highlighted in yellow and contacting residues in red or blue spheres depending from which monomer they originated. (**c**) Steps in the formation of a tightly-packed linear CDC oligomer. PLY was modelled on to the structures of the ILY-CD59 complex and LLO crystal structures by separate superimposition of domains 1 to 3 and domain 4 of PLY on to two adjacent monomers in the ILY and LLO linearly packed crystallographic oligomers. Shown from left to right: the relative orientation of PLY modelled on the ILY-CD59 oligomer, the LLO oligomer and the actual orientation within the PLY oligomer itself. A contraction of the dimer and the straightening of the domains 1, 2, 3 – domain 4 angle occurs in going from left to right.

The monomers in the crystal are tightly packed in an elongated linear arrangement (Fig. 2a). An SDS-AGE gel of the PLY crystals reveals a ladder of high molecular weight SDS-resistant oligomers with monomer and low molecular weight oligomers also evident (Supplementary Fig. S1). The crystals were extremely durable during washing and required boiling in SDS loading buffer for solubilisation. The presence of persistent oligomers in the crystal sample suggests that the bonds between monomers in the crystals are strong enough to be partially resistant to both SDS and heat treatment. The interactions observed between the monomers might reflect how an initial CDC oligomer assembles after membrane binding, prior to forming rings. The buried surface between PLY subunits is extensive, with more than 1500 Å^2^ buried in the interface (750 Å^2^ from one monomer and 770 Å^2^ from the second). The subunit interfaces contain three salt bridges (K18 to E84, D93 to R208 and K188 to E260) with two other potential salt bridges (K171 to D59 and D398 to K442) where the residues are in close proximity but not appropriately oriented. Disruption of two of the three salt bridges between PLY monomers was achieved by substituting K18 or R208 with alanine (Fig. 2b). The resulting mutants exhibited less than 3% native PLY activity, suggesting that these electrostatic interactions are critical components of the monomer-monomer interface (Table 1). There are 13 hydrogen-bonding interactions and 68 van der Waals interactions with the majority of contacts located in domain 1 and 3 (Supplementary Table S1). In total there are 56 residues involved in intermolecular contacts (defined as residues containing atoms within 4 Å of each other) and the shape complementarity score of 0.56 is indicative of specific but weak protein-protein interface^29^. There is also a striking charge complementarity at the interfaces (Fig. 1c).

To further examine residues involved in the monomer-monomer interface, bulky side-chains were introduced in two key positions: N66, which is positioned between T201 and S203 of the neighbouring monomer, and S68, which interacts with D205 of the adjacent monomer (Fig. 2b). Substitution of N66 with tryptophan reduced cytolytic activity by 50%, while substitution of S68 with tryptophan had no affect (Table 1). However, mutation of both residues to tryptophan (N66W∙S68W) abolished activity (Table 1). Furthermore, the introduction of steric bulk combined with disruption of the R208-D93 salt bridge by mutation of R208 to alanine, also eliminated cytolytic activity (Table 1). Taken together, these results indicate that specific monomer-monomer contacts at this interface must occur to drive PLY pore formation.

There are two other CDC crystal structures in which a linear oligomer has been observed in the crystal lattice. In the structure of the ILY:CD59 complex, the ILY molecules interact with each in an extended array in a similar fashion to PLY with a lower shape complementarity score of 0.45^19^. However, the total number of intermolecular contacts (10 vs 56 in PLY), and small buried surface area (140 Å^2^ per monomer) point to weak interactions between monomers. A similar packing is also found in the crystal structure of LLO where there is a shape complementarity score of 0.47, with a total of 16 intermolecular contacts and slightly larger buried surface areas (287 Å^2^ per monomer)^26^. The biological relevance of these interfaces was emphasised by the observation that mutation of residues at the LLO interface (Lys 175 Glu, Glu 262 Lys, Ser 176 Trp) abolished hemolytic activity^26^. These data suggest a possible model for monomer-monomer interactions, which is the first step in the formation of the CDC prepore (Fig. 2c). Initially, a weak interaction forms between the monomers (as seen in the ILY oligomer), with very few contacts and only small buried surfaces at the protein interfaces. The monomers then pack together more tightly, increasing the number of contacts and further burying protein surface (as seen in the LLO oligomer) before they reach a fairly tight oligomer with extensive contacts and significant buried surfaces (as seen in the PLY oligomer). Simultaneously there is a straightening of the D1, 2, 3 – D4 angle as the proteins transition from loosely packed to tightly packed structures (Fig. 2c). When the PLY crystal structure is mapped onto the ILY and LLO oligomers (Fig. 2c), it reveals a core of half a dozen interactions at the PLY monomer-monomer interface that are conserved in all three linear oligomers: Asp 177 to Lys 152, Asn 181 to Arg 51, Gln 225 to Leu 11, Lys 288 to Glu 264 (salt bridge), Ser 298 to Arg 147 (hydrogen bond) and Thr 304 to Lys 268. The interactions in all but one case involve D3 interactions (the exception being one D1–D1 interaction), with the majority of these between D3 of the neighbouring monomer but with one example each of D3 interactions with D1 or D2 (Supplementary Table S1).

### Solution structure of PLY reveals a monomer

**Figure 3:**
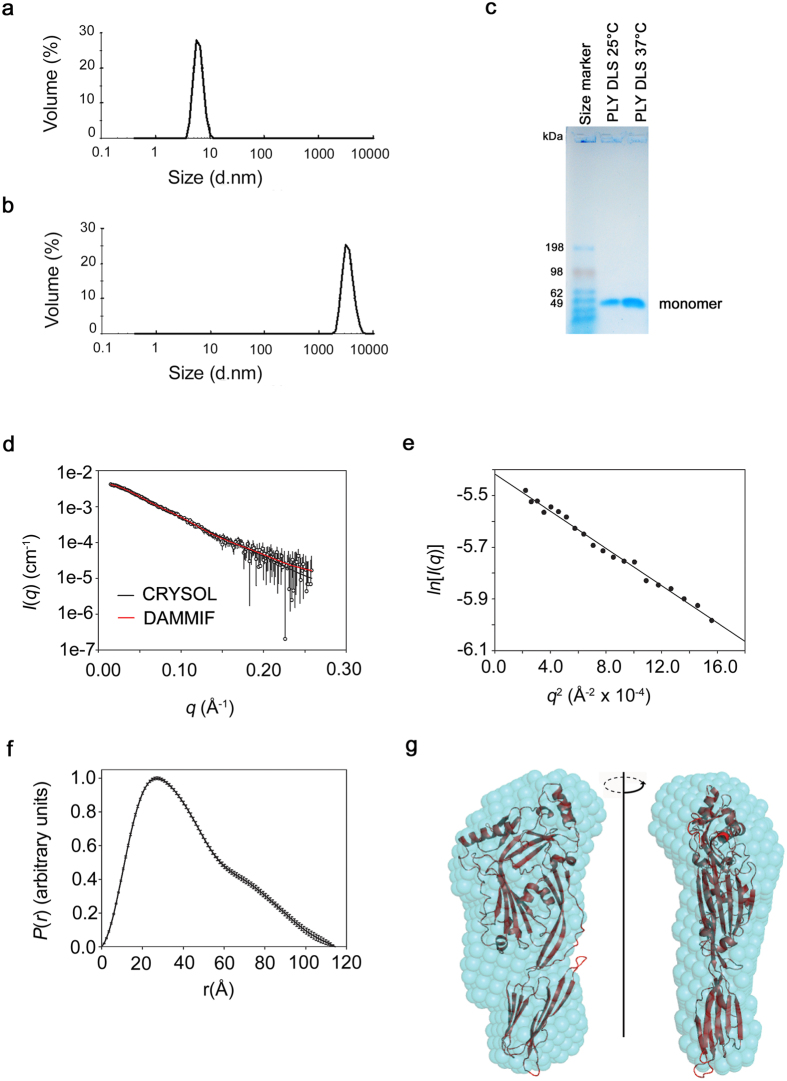
Analysis of PLY oligomers in solution. (**a**) Dynamic light scattering profile after incubation at 25 °C. (**b**) Dynamic light scattering profile after incubation at 37 °C for 10 minutes. (**c**) SDS agarose gel analysis of the same PLY solutions that were examined by DLS. The expected PLY molecular weight is 53.7 kDa based on amino acid sequence. (**d**) SEC-SAXS analysis. The mean intensities as a function of the magnitude of the scattering vector (*I*(*q*) vs. *q*) are shown as circles and the error bars indicate ±1 standard deviation. The theoretical scattering profile of the PLY crystal structure was fitted to the experimental data using CRYSOL^37^ and is shown as a solid black line (Chi_CRYSOL_ = 0.611). The fit of the theoretical scattering profile of a representative dummy atom model generated by DAMMIF^36^ is shown as a solid red line (Chi_DAMMIF_ = 0.593). (**e**) Guinier plot. The first 20 data points of the scattering data satisfied *q*.*R*_g_ < 1.3. (**f**) Pair distance vector distribution function generated from the SAXS data using GNOM^63^. (**g**) Comparison of the SAXS-derived *ab initio* shape envelope with the PLY crystal structure. The averaged filtered shape envelope from DAMAVER^53^, derived from 10 DAMMIF models, is shown in cyan spheres and has been optimally superimposed on the PLY structure. The normalised spatial discrepancy for the fit was 1.21.

The presence of PLY oligomers in the crystal raised the question as to whether they also exist in solution. To answer this question we first subjected PLY (0.2 mg/ml) to dynamic light scattering (DLS) analysis (Fig. 3a,b, Supplementary Table S2). A distinct peak with a mean particle diameter of 6.6 nm was obtained after PLY incubation at 25 °C (Fig. 3a, Supplementary Table S2) that corresponded to a PLY monomer based on a comparison to protein standards. PLY samples incubated at 37 °C for 10 minutes resulted in a narrowly defined peak of particle diameter 3446 nm (±834) (Fig. 3b, Supplementary Table S2) that corresponded to a well defined oligomer. This correlates with a previous report that PLY forms regular sized, ring-shaped oligomers in solution^30^.

The nature of the oligomer at 37 °C was further explored by subjecting both DLS samples to SDS-AGE tricine gels to determine whether the oligomeric structure was SDS-resistant. Samples incubated at both 25 °C and 37 °C ran as monomers (Fig. 3c) indicating that the oligomer formed at 37 °C was not SDS-resistant. Previously, SDS-sensitive PFO oligomers have been characterised in pore formation assays on liposomes^31^. The distinct DLS peak shape of the oligomer suggests a uniform size population, possibly linear or circular oligomers. Soluble PLY oligomers have been reported in the past and shown by electron microscopy to adopt circular structures^30^. It seems unlikely that the oligomers observed in DLS correspond to the linear oligomers observed in the crystals, as one might expect linear oligomers to adopt a broad range of sizes in solution, consistent with previous findings for the linear oligomers formed by mutants of Trp 165 in PFO^32^. A likely explanation as to why the oligomers in solution circularise is that the higher temperature provides enough energy to dislodge D3 from the body of the molecule and thus allows the conformational changes required for the prepore to form^28^.

The nature of the PLY sample that was used for the crystallographic studies was further explored by small-angle X-ray scattering (SAXS) in combination with size exclusion chromatography (SEC-SAXS)^33^. Samples incubated at 37 °C proved poorly soluble for SAXS analysis so only samples incubated at 25 °C were analysed. The scattering species eluted in a single peak with a maximum length (*D*_*max*_) of 115 Å and a radius of gyration of 32.9 ± 0.5 Å (Supplementary Table S3 and Fig. 3d–f). The molecular mass of the scattering species was calculated to be 53.8 ± 0.6 kDa using the volume estimate provided by AUTOPOROD^34^ and the SAXS-derived partial specific volume of 0.7425 cm^3^/g^35^. This correlates with the molecular mass of 53.7 kDa calculated from the PLY sequence and is an indicator that the molecule is monodisperse and monomeric in solution. The shape of the *P*(r) curve (Fig. 3f) suggests a slightly elongated molecule rather than a completely globular molecule (Fig. 3e). An *ab initio* elongated and relatively flat shape envelope of PLY (Fig. 3g) was calculated from the *P*(*r*) distribution using dummy atom modelling^36^. The crystallographic coordinates of PLY fit into the envelope well and, as expected, the theoretical scattering profile generated from the coordinates of PLY using CRYSOL^37^ fitted the experimental SAXS scattering profile well (Fig. 3d). In summary, the SAXS data reveal that PLY (at 25 °C) adopts a monomeric structure that is very similar to the structure observed by crystallography.

### *In silico* docking of sLeX, LeB and mannose to PLY

**Figure 4:**
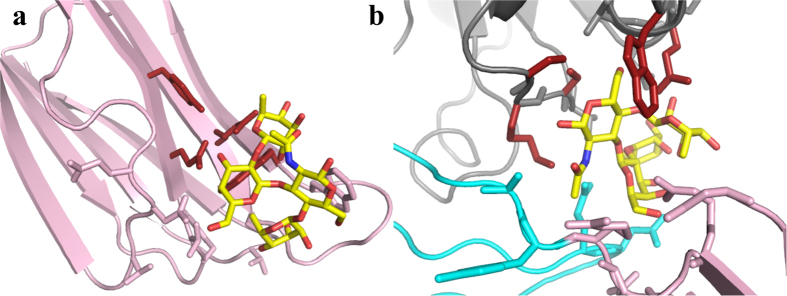
Potential carbohydrate binding sites in PLY. In both sites the best docked pose for sLeX is shown as yellow sticks coloured by atom type. The protein is coloured by domain as in Fig. 1 and shown as cartoon, with the predicted carbohydrate-binding residues shown as sticks coloured red. The rest of the carbohydrate-interacting residues are shown as sticks coloured by domain. (**a**) Site 1. (**b**) Site 2.

Building on work that suggested a role for mannose in the regulation of PLY oligomerisation^21^, it was suggested that PLY might use certain carbohydrate moieties from glycolipids and glycoproteins as receptors on the membrane surface of host cells^20^. The glycan sialyl Lewis X (sLeX) bound with the highest affinity (*K*_d_ = 1.88 × 10^−5^M) to PLY whereas the related Lewis B (LeB) did not bind at all^20^. A carbohydrate binding prediction server^38^ was used to identify potential glycan binding sites on a homology model of PLY that predicted a binding site in D4. Site-directed mutagenesis studies corroborated that the D4 residues Gln 374, Tyr 376, Trp 433 and Leu 460 were involved in binding sLeX^20^. However, a comparison of the PLY-derived homology model used for the prediction to the PLY crystal structure reported here suggests there are significant differences in these structures, particularly in the location of residues in D4 such as Trp 433. Thus we used the same prediction server on the PLY crystal structure and have identified two possible binding sites: Site 1 (Gln 374, Tyr 376, Arg 426, Trp 436) corresponding to the published prediction in D4 and Site 2 (Ser 254, Lys 255, Ser 256, Trp 278, Gln 280), at the D3-D4 interface (Fig. 1a). We then computationally docked sLeX, LeB and mannose at both sites using Sybyl-X 2.1 (Certara, L.P.; http://tripos.com/) (Fig. 4). The docking scores predicted that Site 2 was a much more favourable binding site for all three compounds than Site 1 (Supplementary Table S4). Both glycans sLeX and LeB can bind nearly as well at both sites, with Site 1 preferring LeB and Site 2 preferring sLeX. Mannose is a significantly weaker binding ligand at both sites.

## Discussion

We have crystallised and solved the structure of PLY and showed that the overall structure of PLY is globally similar to the structure of other CDCs but does contain differences in variable loop regions. The molecule was more elongated compared to other CDCs with no bend in the angle between the main body of the molecule (D1-3) and D4. This straightening of the PLY molecule may be the result of PLY lacking the restraints that other CDCs experience from the impact of hydrogen bonds that are not existent in PLY at the D2-4 interface. The structure determined by SAXS is consistent with PLY maintaining its linearity in solution (Fig. 3), consistent with old studies suggesting PLY is an elongated molecule in solution^39^. Early electron microscopy studies suggested PLY might adopt a bent shape when it oligomerises^40^ but more recent studies indicate that rather than bending, the toxin undergoes a substantial tilt relative to the membrane surface^28^. Our structural studies have also shown that the Trp-rich loop of PLY adopts a conformation similar to SLY and SLO.

Our studies further suggest a structure for the early interactions of CDC monomers at the membrane surface, which is consistent with published results^32^,^41^. It is striking that the three CDCs shown to form linear oligomers in their crystalline state cluster together in a minor clade of CDCs that lack the components of the electrostatic switch^28^. The analogous residues to the components of the electrostatic switch in PFO (Glu 183, Lys 336) are not conserved in ILY (Ser, Gly), LLO (Asp, Asp) or PLY (Lys, Gly).

The mutation of selected residues within the interface guided by the crystal structure of the linear oligomer revealed that all residues either individually or in combination resulted in significant effects on PLY pore-forming activity. Both basic residues, K18 and R208, which participate in electrostatic interactions with E84 and D93, respectively, at the monomer interface decreased hemolytic activity ≥100-fold. Interestingly, mutants where N66 or S66 were substituted with tryptophan retained near wild-type activity; however, in combination they reduced activity >100-fold. These studies strongly suggest that these residues contribute to the assembly of functional pores, presumably by preventing or altering the correct intermolecular interactions between monomers at the membrane surface.

**Figure 5:**
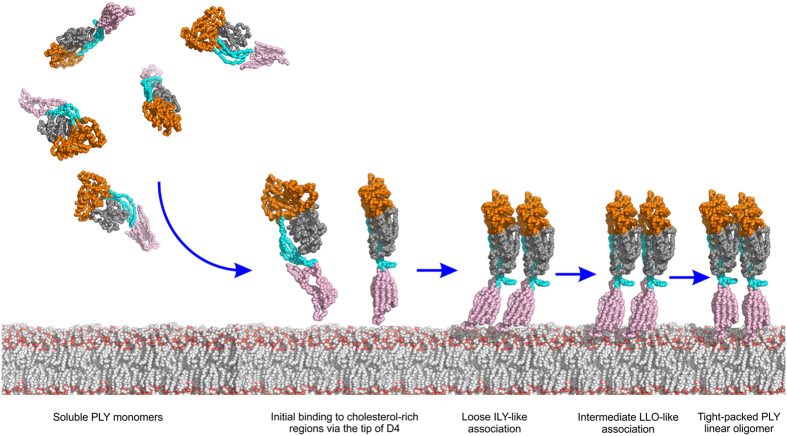
Schematic showing early stages of PLY oligomerisation based on the PLY crystal structure and mapping of the PLY crystal structure on the ILY-CD59^19^ and LLO^26^ structures. PLY is shown as a simplified bead model, coloured by domain as in Fig. 1. PLY approaches the target membrane as soluble monomers and binds to cholesterol-rich regions via the loops at the tip of domain 4. Monomers start to form a loose linear assembly, primarily via domains 1 and 3, as observed in the PLY model based on ILY. As packing between the monomers becomes tighter, the interactions increase to reflect those seen in PLY model based on LLO. Finally, the monomers become tightly packed resembling the linear SDS-sensitive oligomer as seen in the PLY crystal structure. All these linear oligomers are transient in nature but can be trapped by mutation (see text) or in the crystal. In the later stages of cytolytic activity (not shown) interactions with cholesterol trigger conformational changes in domain 3 leading to tilting of the monomers, oligomer circularisation and an SDS-sensitive early prepore. Structural rearrangements in domain 3 lead to the SDS-resistant late prepore before unfurling of the TMH regions in domain 3 to form the SDS-resistant pore (not shown).

CDC linear oligomers have previously been observed on membranes with certain mutants of PFO^32^. Mutation of the D1 residue Trp 165, which is conserved in all CDCs (Trp 134 in PLY), to non-aromatic residues inactivates PFO by preventing all D3 structural transitions, which results in the formation of linear membrane oligomers. Deletion of the nearby residue Ala 146 in PLY (Ala 177 in PFO) also decreases pore-forming activity and results in the formation linear membrane oligomers^41^. Native PFO assembles SDS-resistant oligomers as judged by SDS-AGE, but the non-aromatic mutants for Trp 165 form SDS-sensitive linear oligomers that are trapped in an early transient intermediate stage in which monomers have begun to assemble on the membrane surface. In wild-type toxin these interactions would promote several conformational changes, including a 30° tilt of D1-3 with respect to D4 towards the membrane, which would propagate throughout the growing oligomer to establish the geometry of the ring complex and irrevocably commit membrane-bound monomers to the formation of the oligomeric pore structure. It is likely that concentrating these PFO and PLY mutants on the membrane surface or concentrating PLY during crystallisation allowed the monomers to initiate these interactions. Thus it seems the linear oligomers observed in the ILY, LLO and PLY structures are glimpses of the very early stages of weaker monomer-monomer interactions required before the interactions become strong enough to drive the conformational changes needed to induce circularisation of the oligomer. A schematic showing these early stages of CDC oligomerisation is presented in Fig. 5.

The fact, however, that the monomers only form linear oligomers during crystallisation shows that a membrane interaction is required to initiate changes in the monomer structure in order to alter the geometry of this interaction to form the circular oligomers^13^. A key structural change to establish the circular geometry of the oligomer is the displacement of β-strand 5^32^. It is apparent from the crystal structure of the linear complex that β-strand 5 remains in place. Its disengagement from β-strand 4 is necessary for the subsequent formation of backbone hydrogen bonds with β-strand 1 of the adjacent monomer within the oligomeric complex^3^. By preventing the interaction between these β-strands the final geometry of the monomer-monomer interaction is not established, which prevents the formation of circular complexes, thereby resulting in the linear oligomeric structure^32^.

Our docking studies also suggest two possible binding sites for the recently identified LeX and sLeX glycans as putative receptors for PLY^20^: one near the conserved undecapeptide close to the base of D4, similar to that previously suggested for a PFO-based PLY model^20^ and a second one at the D3-D4 interface, which is at the top of D4. If PLY does bind to a LeX and sLeX glycan receptor prior to forming a cholesterol-dependent interaction with the membrane then the second site identified herein seems more likely to properly orient the cholesterol-binding motif at the membrane surface. However, it remains to be shown unambiguously that PLY uses LeX or sLeX as a receptor. PLY crystals soaked in mannose (a thousand-fold excess) or co-crystallised with the toxin did not reveal bound glycan; however, it is not possible to soak the crystals at the ≥million-fold molar excess of glycan over PLY that was reported to be necessary for inhibition of PLY binding and hemolytic activity^20^. At the excessive sLeX to PLY ratio used to suggest sLeX as a receptor for PLY it cannot be ruled out that this particular glycan, at the very high relative concentration utilised, could have fortuitously interacted in a non-specific manner with the undecapeptide tryptophans: tryptophan is the most frequently preferred residue in carbohydrate binding proteins^42^. Binding to the undecapeptide could have inhibited binding and hemolytic activity in a manner unrelated to acting as a receptor by altering the structure of D4 as it is well known that the CDC mechanism is highly sensitive to changes in the undecapeptide structure^13^,^43^,^44^,^45^.

New protein-based pneumococcal vaccines are increasingly being investigated to eliminate the necessity of capsule-based vaccines, which exhibit >90 different variations, and PLY has been deemed an important component for these future vaccines due to its highly conserved structure and importance to *S. pneumoniae* virulence^41^,^46^,^47^,^48^,^49^,^50^,^51^. Therefore, the structure of PLY provides an important basis for the design of new treatments, including both small molecule drugs and vaccines, to treat pneumococcal diseases. The structure of the PLY oligomer provides the basis from which experiments can be designed to better understand the conformational changes and molecular interactions that are formed as the CDCs make the transition from the early stage of monomer-monomer interaction at the cell surface to the formation of the stable pore complex.

## Methods

### Cloning, expression and purification

Wild-type PLY was cloned into pQE-30^9^. Hexahis-PLY (referred to as PLY herein) was expressed in *E. coli* BL21 (DE3) pREP4 in Turbo Broth medium (Athena Enzyme systems, Baltimore) at 37 °C. The cell pellet was resuspended at 21 °C in lysis buffer (25 mM Tris-HCl, 500 mM NaCl, 10% (v/v) glycerol, 0.1% (v/v) Triton X-100, 100 μM PMSF, 10 μg/ml lysozyme, 10 μg/ml DNAse 1, 20 mM imidazole, pH 7.2). Cells were lysed at 21 °C by shaking them for 1 hour, then sonicated 6 × 10 seconds. PLY was purified on a 5 ml HisTrap^TM^ HP column (GE), equilibrated in Buffer A (25 mM Tris-HCl, 500 mM NaCl, 20 mM imidazole, 10% glycerol, pH 7.2). The column was washed with 50 ml Buffer A, pH 7.2. PLY was eluted from the column with Buffer B (25 mM Tris-HCl, 500 mM NaCl, 400 mM imidazole, 10% glycerol, pH 7.2). Fractions containing PLY were pooled and dialysed into 20 mM HEPES, 150 mM NaCl, pH 7.5 at 21 °C for 16 hours. Dialysed PLY was further purified by size exclusion chromatography on a Superdex 200 26/60 column (GE) in 20 mM HEPES, 150 mM NaCl, pH 7.5. Fractions containing PLY protein (monitored by SDS-PAGE) were pooled and concentrated to 10 mg/ml and stored at −80 °C.

Residues within PLY that were predicted to mediate monomer-monomer interactions were mutated by PCR QuickChange mutagenesis (Stratagene) using codon-optimised PLY as the template. The Laboratory for Molecular Biology and Cytometry Research at the University of Oklahoma Health Sciences Center performed sequencing to verify incorporation of the mutations. PLY and PLY derivatives were transformed into *E. coli* Tuner cells and then purified as described^5^. Protein concentration was assayed using Protein Assay Dye Reagent Concentrate (Bio-Rad) and extrapolated from a standard curve using BSA.

### Dynamic light scattering

Dynamic light scattering (DLS) was carried out using a Malvern Instruments Zetasizer Nano series instrument. Samples were assayed in Greiner bio-one polystyrene semi-micro cuvettes with a path length of 10 mm. Data were analysed using Zetasizer Version 7.02 software. PLY was assayed at a concentration of 0.2 mg/ml in a 300 μl volume in the presence of 150 mM NaCl, 10 mM Tris-HCl pH 7.5 and 2 mM EDTA. Samples were incubated at 25 °C or 37 °C for 10 minutes and centrifuged at 13,000 × g for 3 minutes, prior to loading. Three technical replicates were measured for each sample. The mean for the technical replicates was calculated, then the means of the technical replicates were combined to determine the mean of *n* = 3. The particle diameter (nm) is reported from intensity analysis and the peak relative percentage is reported from the volume distribution analysis.

### Thermal melting temperature (T_m_) of PLY

The T_m_ was determined as previously described for PFO^28^.

### Cytolytic activity of PLY and PLY derivatives

The cytolytic activities were determined as described for PFO on human red blood cells (RBCs)^5^. The effective concentration required for 50% cytolysis (EC_50_), calculated from a non-linear sigmoidal dose-response curve fit of the data, as well as the standard error of the mean, are reported.

### SDS agarose gel analysis

Six times non-reducing SDS loading buffer (2 μl) was added to 20 μl (0.2 mg/ml) PLY samples from the DLS assays described above and were run on a 1.5% SeaPlaque® agarose (Lonza) SDS agarose gel (SDS-AGE) tricine gel in tricine buffer (25 mM Tris–HCl, 250 mM glycine, 1% SDS, pH 8.3) at 110 mA for 1 hour. The gel was partially dehydrated and stained with Instant Blue stain (Expedeon) and destained in 7% acetic aid, 10% methanol.

PLY crystals (not grown in the presence of the gold compounds) scooped from a 4 μl volume crystallisation hanging drop were washed with 100 μl of 20 mM HEPES, 150 mM NaCl, pH 7.2 and collected by centrifugation. The washing was repeated three times. Residual wash solution was removed and crystals were solubilised in 20 μl wash buffer and 4 μl 6 × SDS, non-reducing, loading buffer (375 mM Tris-HCl pH 6.8, 6% SDS, 48% glycerol and 0.03% bromophenol blue). The protein were boiled for 2 minutes at 95 °C and loaded onto SDS-AGE gel with 10 μl SeeBlue® Plus2 Pre-stained Protein Standard (Life Technologies) and 1 μg PLY monomer as size references.

### Small-angle X-ray scattering analysis

Small–angle X-ray scattering (SAXS) data were collected on the SAXS/WAXS beamline at the Australian Synchrotron (Clayton, Victoria). The X-ray beam size was 250 μm horizontal × 120 μm vertical. Data collection was on a Dectris-Pilatus 200 K detector. Sample to detector distance was 1576 mm (*q*-range 0.006–0.4 Å^−1^) with a 11 Ke V beam and X-ray wavelength of 1.0322 Å. PLY (80 μl at 5.35 mg/ml in 20 mM Tris-HCl pH 7.5, 150 mM NaCl and 1 mM DTT) samples were run using in-line gel filtration^33^ with a Wyatt Technology WTC-050N5G SEC column equilibrated in the same buffer. The column flow rate was 0.8 ml min^−1^ at 298 K and exposures were 2 seconds (2.1 sec. repeat time). Data acquisition and reduction analysis were carried out with Australian Synchrotron scatterBrain 9-1_0 software^52^. Further analysis was carried out using ATSAS 2.6.0 data analysis software^34^. *Ab initio* shape reconstructions were performed using *DAMMIF*^36^. Averaged filtered shape envelopes were generated from ensembles of 10 *DAMMIF* envelopes with *DAMAVER*^53^. Theoretical scattering profiles were generated from model coordinates and compared to experimental data using *CRYSOL*^37^. Dry volume was calculated using http://www.basic.northwestern.edu/biotools/proteincalc.html.

### Crystallisation

Initial crystallisation trials of PLY were set up at 21 °C on a Crystal Gryphon robot (ARI, Sunnywale CA, USA) in 96-well sitting drop format using Rigaku UV + 96 plates (AXT, Sydney, Australia). Drops containing 0.2 μl of protein and 0.2 μl of crystallisation solution were equilibrated against 35 μl of crystallisation solution. PLY was in 150 mM NaCl, 0.5 mM DTT, 20 mM Tris-HCl pH 7.5 at a concentration of 10.5 mg/ml. Crystallisation screens were set up at room temperature and included MCSG-I, MCSG-II, MCSG-III and MCSG-IV from Microlytic (Burlington, USA). The screen solutions gave a number of initial hits in drops of solutions 23, 37, 38 of MCSG -III and 4, 24, 31, 44, 65 in MCSG-IV, resulting in needle and plate crystals. The drops were scaled up to drops containing 2 μl protein and 2 μl precipitant solution in a hanging drop format using Linbro culture plates (ICN, Biochemicals Inc., Ohio, USA) and equilibrated against 1 ml of well solution. The best needle crystals grew in solution 37 of MCSG-III. Improvement of the size of the needles was achieved by using a range of different concentrations of precipitant and a range of buffer pHs. A shower of rod crystals each of a size of 0.02 mm × 0.02 mm × 0.6 mm grew within a day in 10% polyethylene glycol (PEG) 8000, 10% glycerol, 10% ethylene glycol and 100 mM HEPES buffer at pH 7.5. Flash freezing was performed by transferring the crystals straight from the drop into liquid nitrogen. These crystals diffracted to ~3.6 Å resolution but initial phases derived from molecular replacement approaches were of poor quality (see below) prompting a search for heavy atoms to improve the phases. In one experiment 5 mM KAu(CN)_2_ was made up in the crystallisation buffer and mixed with PLY. The addition of the heavy atom was crucial for the growth of larger crystals (typically 0.04 mm × 0.04 mm × 0.8 mm) and resulted in better diffraction of 2.9 Å resolution.

### Data collection, structure determination and refinement

X-ray diffraction data were collected at the Australian Synchrotron Beamline MX2 (Clayton, Australia) at 100 K using the program Blue-Ice^54^. The data were processed with the program XDS^55^. The crystals belonged to the space group *P2*_*1*_*2*_*1*_*2*_*1*_ with unit cell dimensions of *a* = 24.9 Å, *b* = 133.6 Å and *c* = 220.4 Å. One molecule was found in the asymmetric unit giving a V_M_ value^56^ of 3.46 Å Da^−1^ and an estimated solvent content of 64%. Molecular replacement was first used to obtain initial phases. Initial attempts with PFO (intact and by domain) proved fruitless. It was then decided to try the crystal structure of SLY (PDB code: 3HVN) as a probe since it is the CDC of known 3D structure with the most similar sequence to PLY (sequence identity of 52%). A molecular replacement solution was obtained but the resultant phases were of poor quality. A heavy atom search led to the discovery that co-crystallisation of PLY with KAu(CN)_2_ resulted in the growth of larger crystals (typically 0.04 mm × 0.04 mm × 0.8 mm) and to an increase in diffraction from 3.6 Å to 2.9 Å resolution. With the better quality data the molecular replacement approach using SLY was much more successful. Molecular replacement was performed using the program Phaser^57^ with domains 1 to 3 and domain 4 of SLY as two separate ensembles. Alternate rounds of model building and refinement were carried out with the program COOT^58^ and PHENIX^59^. Electron density for three N-terminal residues, all part of the Hexhis tag/linker region, were visible and added to the model. Twenty-nine water molecules, 5 ethylene glycol molecules, two PEG molecules (consisting of two ethylene glycol units) and two gold cyanide compounds (occupancies of 0.4 each) bound to Cys 428 were built into the structure. The final model of PLY resulted in *R*_work_ and *R*_free_ of 19.9% and 30.7%, respectively. The data and refinement statistics are given in Supplementary Table S5. A Ramachandran plot produced by PROCHECK^60^ showed that 97.7% are in the allowed regions and there are 10 outliers (Phe 32, Glu 35, Lys 34, Ala 107, Glu 189, Ile 267, Met 309, Asp 311, Thr 321 and Lys 389). All are in regions of poor density.

### Prediction of carbohydrate binding sites and computational docking

Residues identified by the carbohydrate binding prediction server^38^ were used as the basis for defining a pair of Surflex binding sites using Sybyl-X 2.1 (Certara, L.P.; http://tripos.com/). This process included a set of additional residues that defined a larger potential binding surface than that identified as ‘likely sugar contacts’ by the server. For Site 1 these were Tyr 371, Val 372, Gln 374, Tyr 376, Gly 401, Gln 402, Asp 403, Thr 405, Arg 426, Cys 428, Trp 435, Trp 436 and Thr 459. For Site 2 these were Asp 41, Glu 42, Thr 253, Ser 254, Lys 255, Ser 256, Trp 278, Gln 280, Ile 281, Asp 283, Asn 284, Ala 357, Tyr 358, Arg 359, Asn 360, Gly 361, Arg 449 and Asn 470. Mannose, LeB and sLex were built in SybylX-2.1 and docked into both putative carbohydrate binding sites using Surflex^61^ in GeomX mode. Docked poses for each compound were inspected in SybylX2.1.

## Additional Information

**Accession codes**: Coordinates and structure factors have been deposited in the Protein Data Bank under the accession code 4ZGH.

**How to cite this article**: Lawrence, S. L. *et al.* Crystal structure of *Streptococcus pneumoniae* pneumolysin provides key insights into early steps of pore formation. *Sci. Rep.* **5**, 14352; doi: 10.1038/srep14352 (2015).

## Supplementary Material

Supplementary Information

## Figures and Tables

**Table 1:** Cytolytic activity of PLY derivatives with mutations in the monomer-monomer interface.

**Toxin**	**EC_50_ (nM)**	**% Native PLY activity**
PLY	0.013 ± 0.004	100.00
PLY^K18A^	ND	ND
PLY^R208A^	0.52 ± 0.07	2.50
PLY^N66W^	0.026 ± 0.01	50.00
PLY^S68W^	0.013 ± 0.005	100.00
PLY^N66W∙S68W^	2.03 ± 0.47	<1.00
PLY^N66W∙S68W∙R208A^	2.98 ± 0.66	<1.00

The mean effective concentration (EC_50_; the concentration of toxin required to lyse 50% of RBCs) and the standard error of the mean from a minimum of three experiments are shown for each toxin. The percentage cytolytic activity of each mutant relative to native PLY activity [(EC_50_ native PLY/EC_50_ mutant) × 100] is also shown. ND, not determined because an EC_50_ could not be calculated as the highest concentration of toxin did not achieve 100% hemolysis.
