# Supplementary material for: Crystal structure of Streptococcus pneumoniae pneumolysin provides key insights into early steps of pore formation
Source: Sci Rep. 2015 Sep 25;5:14352. doi: 10.1038/srep14352 (PMC4585913; doi:10.1038/srep14352)
Supplement: Supplementary Information [file srep14352-s1.pdf]

Crystal structure of *Streptococcus pneumoniae* pneumolysin provides key insights into early steps of pore formation

Sara L. Lawrence[1*], Susanne C. Feil[1*], Craig J. Morton[1*], Allison J. Farrand[2*], Terrence D. Mulhern[3], Michael A. Gorman[1], Kristin R. Wade[2], Rodney K. Tweten[2] & Michael W. Parker[1,2]

[1]ACRF Rational Drug Discovery Centre, St. Vincent's Institute of Medical Research, Fitzroy, Victoria 3065, Australia, [2]Department of Microbiology and Immunology, University of Oklahoma Health Sciences Center, Oklahoma City, Oklahoma 73104, USA, [3]Department of Biochemistry and Molecular Biology, Bio21 Molecular Science and Biotechnology Institute, The University of Melbourne, Parkville, Victoria 3010, Australia.

*Equal first authors

Correspondence and requests for materials should be addressed to M.W.P. (mparker@svi.edu.au)

Supplementary Figures

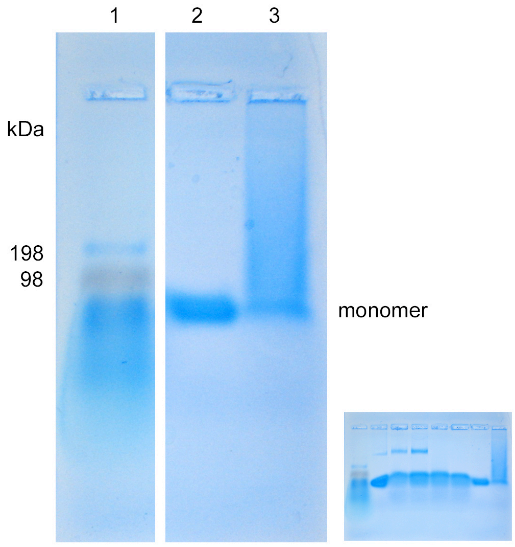

Figure S1. SDS-AGE gel of SDS and heat solubilised PLY crystals. PLY crystals were solubilised in SDS non-reducing buffer and boiled (lane 3), prior to loading onto a SDS-AGE gel (1.5% agarose/ Tricine). Protein standard size marker SeeBlue® Plus2 (lane 1) and 1µg solution of PLY monomer (lane 2) were run for comparison. The PLY crystal sample shows a continuous size population of PLY species from monomer to high molecular oligomer. The whole gel from which these lanes were derived is shown in the inset.

Supplementary Tables

Supp. Table S1. Intermolecular contacts in linear oligomers of PLY, ILY and LLY

Protein	Contacts (Monomer 1 : Monomer 2)
ILY	R127 (D3) : I399 (D1)<br>L144 (D1) : I399 (D1)<br>K229 (D3) : I399 (D1)<br>N235 (D3) : D322 (D3) (HB)<br>V360 (D3) : K326 (D3)<br>C361 (D3) : K326 (D3)<br>H465 (D4) : R495 (D4) (HB)<br>Y466 (D4) : R495 (D4)<br>T468 (D4) : L496 (D4)<br>K473 (D4) : D502 (D4) (SB)
LLO	A120 (D1) : L397 (D1)<br>L124 (D1) : E396 (D1)<br>E136 (D1) : N73 (D1) (HB)<br>Q141 (D1) : N73 (D1)<br>V144 (D1) : E396 (D1)<br>K175 (D1) : E262 (D1) (SB), N395 (D1)<br>S176 (D1) : P41 (D1)<br>N230 (D3) : N109 (D2) (HB), Q110 (D2)<br>D354 (D3) : K203 (D1)<br>E355 (D3) : K203 (D1), I204 (D3) (HB)<br>L461 (D4) : W489 (D4)<br>Y469 (D4) : D497 (D4), R499 (D4)
PLY	N66 (D1) : S239 (D1)<br>S68 (D1) : D205 (D1), A206 (D1) (HB)<br>R69 (D1) : N339 (D1), V341 (D1)<br>T81 (D1) : K17 (D1)<br>N86 (D1) : V340 (D1) (HB), V341 (D1) (HB)<br>T88 (D1) : D338 (D1), V340 (D1)<br>L89 (D1) : D338 (D1)<br>A91 (D1) : R208 (D1)<br>V92 (D1) : R208 (D1)<br>D93 (D1) : R208 (D1) (SB)<br>N120 (D1) : D205 (D1)<br>S121 (D1) : F112 (D1) , D205 (D1) (HB)<br>R124 (D1) : F112 (D1)<br>G125 (D1) : S108 (D1)<br>N128 (D1) : S108 (D1)<br>E170 (D3) : S56 (D2)<br>K171 (D3) : S58 (D1), D59 (D1)<br>N174 (D3) : T55 (D2) , S56 (D2), T57 (D2), E151 (D3)<br>S175 (D3) : E151 (D3)<br>D177 (D3) : Y150 (D3), E151 (D3), K152 (D3)<br>N181 (D3) : R51 (D2)<br>Q225 (D1) : L11 (D1), D8 (D1) (HB)

	R226 (D1) : L11 (D1), N209 (D1) (HB)<br>E286 (D3) : A265 (D3)<br>K288 (D3) : Y150 (D3), E264 (D3) (SB)<br>P296 (D3) : R147 (D1)<br>S298 (D3) : D102 (D1) (HB), R147 (D1) (HB)<br>A300 (D3) : Q149 (D3)<br>R301 (D3) : R147 (D1), M148 (D3)<br>V302 (D3) : M148 (D3) (HB), Q149 (D3), Y150 (D3)<br>T304 (D3) : K268 (D3)<br>G305 (D3) : K268 (D3)<br>M309 (D3) : K268 (D3), V270 (D3)<br>D398 (D4) : K442 (D4), T443 (D4)<br>R399 (D4) : E441 (D4) (HB), K442 (D4)<br>Q402 (D4) : E441 (D4) (HB)

D1 – domain 1, D2 – domain 2, D3 – domain 3, D4 – domain 4, HB – hydrogen bond, SB – salt bridge

Supp. Table S2. Dynamic light scattering data

Sample	Temperature (°C)	*n*	Particle Diameter (nm)	Standard deviation (nm)	Peak (%)
PLY	25	3	6.6	0.9	99.6
PLY	37	3	3445	833	100

Supp. Table S3. SAXS data collection and scattering-derived parameters

Data-collection parameters	
Instrument	Australian Synchrotron SAXS/WAXS beamline with in-line size exclusion chromatography
Beam geometry	Point collimation (250 μm horizontal × 120 μm vertical)
Wavelength (Å)	1.03320
q range ($Å^{-1}$)	0.0149-0.2583 (189 data points)
Exposure time (s)	41 × 2
Concentration (mg/ml)	0.08
Temperature (K)	298
Structural parameters[a]	
$I(0)$ (cm^{-1}) [from $P(r)$]	0.00450 ± 0.00003
R_g (Å) [from $P(r)$]	34.5 ± 0.4
$I(0)$ (cm^{-1}) (from Guinier)	0.00444 ± 0.00004
R_g (Å) (from Guinier)	32.9 ± 0.5
D_{max} (Å)	114.5 ± 3[b]
Porod volume estimate ($Å^3$)	72489 ± 787
Dry volume calculated from sequence ($Å^3$)	64005
Molecular mass determination[a]	
Molecular mass Mr [from Porod volume] (kDa)	53.8 ± 0.5[c]

[a] Reported for the averaged SAXS data
[b] D_{max} is a model parameter and uncertainty is based on $P(r)$ analyses with a range of D_{max} values
[c] Calculated from the Porod volume using a partial specific volume of 0.7425 cm^3g^{-1} by the method of Mylonas & Svergun[1].

Supp. Table S4. Carbohydrate docking scores[a]

	Sialyl-Lewis X	Lewis B	Mannose
**Site 1**	5.95	9.24	2.06
**Site 2**	13.61	10.14	6.22

[a] Scores reported are the negative of the Surflex score, which is a theoretical K_D value for formation of the docked complex (Surflex manual, www.certara.com).

Supp. Table S5. Crystallographic data and refinement statistics

A. *Data collection statistics*

Temperature (K)	100
Space group	$P2_12_12_1$
Cell dimensions (Å)	
a	24.9
b	133.6
c	220.5
Resolution (Å)	2.9
No. observations	105956
No. unique reflections	17539
Redundancy	6.0 (6.1)
Data completeness (%)	99.4 (96.6)
I/σ_I	12.2 (3.2)
R_{pim} (%)	6.9 (30.2)
CC(1/2)	0.995 (0.897)
R_{merge} (%)	15.7 (69.3)

B. *Refinement statistics*

Non-hydrogen atoms	
Protein	3744
Water	29
Ligands	39
Resolution (Å)	2.9
R_{work} (%)	19.9
R_{free} (%)	30.7
r.m.s deviations from ideality	
Bond lengths (Å)	0.02
Bond angles (°)	1.9
Average *B*-factor ($Å^2$)	
Main-chain	68.6
Side-chain	68.9
Water	40.0
Residues in the allowed regions of the Ramachandran plot (%)	97.7

The values in parentheses are for the highest resolution bin (approximately 0.1 Å width).

References

1. Mylonas, E. & Svergum D.I. Accuracy of molecular mass determination of proteins in solution by small-angle X-ray scattering. *J. Appl. Cryst.* **40**, s245-s249 (2007).
